# Immune challenge of female great tits at nests affects provisioning and body conditions of their offspring

**DOI:** 10.1007/s10211-017-0265-4

**Published:** 2017-05-21

**Authors:** Emilia Grzędzicka

**Affiliations:** 1 Institute of Environmental Sciences, Jagiellonian University, Gronostajowa Street 7, 30-387 Kraków, Poland; 2 Institute of Nature Conservation, Polish Academy of Sciences, Al. Mickiewicza 33, 31-120 Kraków, Poland

**Keywords:** Breeding attempt, Parental investment, Sheep red blood cells, Nestlings, Caterpillars, Spiders, Phytohaemagglutinin (PHA) injection

## Abstract

The trade-off between animal’s parental reproductive effort and survival is still poorly understood. Parental allocation between the workload during breeding attempts and the parents’ own body conditions can be assessed through the offspring quality. Here, I questioned whether the immune responsiveness of female great tits may be considered as a mediator of this trade-off. Specifically, I tested whether (1) the parental reproductive effort decreases, (2) the food composition provided to chicks changes, and (3) whether the nestling immunocompetence and body mass decrease after experimental immunisation. Two populations of great tit *Parus major* occupying nest boxes were studied in Niepołomice Forest and Krzyszkowice Forest (Southern Poland) in 2011 and 2012, respectively. Three days after hatching, half of the females were challenged with sheep red blood cells (SRBC), while other females were injected with phosphate-buffered saline PBS (control). Six days later, food provided by the parents was collected from nestlings. After another 2 days, the offspring’s body mass was measured and wing web swelling in response to an additional phytohaemagglutinin (PHA) injection. In both years, immunocompetence and in 2012 also body mass in the offspring of SRBC-immunised mothers were lower than in control nestlings, indicating a cost of mounting the immune response in the female. Six days after the start of the female treatment, the number of caterpillars and the volume of food items provided by parents to chicks were higher, whereas the number of spiders was lower in nests with SRBC treatment than in control ones. This might be explained by compensational parental feeding after recovery from the inflammation of a female. Thus, the trade-off between parental effort and survival of parents is mediated by the costs incurred for their immunity and can be assessed by the amount and quality of food provided to the nestlings and the offspring condition.

## Introduction

The trade-off between parental reproductive effort and survival may lead to negative fitness consequences for adults (Williams 1966; Trivers 1972; Dijkstra et al. 1990; Stearns 1992). The basis for this relationship is, however, still poorly understood. Survival is an attribute of fitness, which may be difficult to quantify. In contrast, reproduction is a clearly defined physiological event with a starting and an ending point, which can be evaluated by measuring parental effort and costs (Lochmiller and Deerenberg 2000). Previous explanations of the trade-off focussed on physical deterioration during breeding attempts (Drent and Daan 1980) or accelerated senescence of an organism (Partridge 1978). Physiological explanations assume that reproduction and survival compete for limited energy and nutrients (Harshman and Zera 2007). However, external factors may also interact with this trade-off. Increased predation risk of parents, protecting offspring, as well as frequent infections during the breeding season may affect the parents’ survival (Magnhagen 1991; Gustafsson et al. 1994; Saino et al. 1999).

It is worth emphasising that immunity is one of the major physiological mechanisms, which determine host survival (Lochmiller and Deerenberg 2000). The immune system is a defence mechanism to control and fight any parasitic or pathogenic infection, and thus moderates interactions with the environment (Sheldon and Verhulst 1996; Zuk 1996), and it also carries costs (Bonneaud et al. 2003). Therefore, immune response is often involved in trade-offs with other demanding traits (Sheldon and Verhulst 1996; Ilmonen et al. 2000; Norris and Evans 2000). When the availability of resources required for reproduction and efficient immune response is limited, then natural selection leads to a resource allocation in adults (Williams 1966; Stearns 1992). Parental allocation between workload during breeding attempts and own immune response can be assessed through the offspring condition, which is a key determinant of fitness (Nager et al. 2000; Tella et al. 2000). Research on the parental immune response and its association with offspring conditions may contribute to understand the trade-off between parental effort and survival.

Unexpected stressful events affect the individual’s body conditions and its food intake (Harding et al. 2009; Tilgar et al. 2010). When parents are forced to shift the allocation of nutrients from the immune system and self-maintenance to raising offspring, this will reduce immune function and may affect parental survival. Experimental activation of the immune system results in additional fitness costs (Hasselquist and Nilsson 2012). Klasing (1998) stated that the immune system is a significant consumer of nutritional resources. Fighting an infection results in reduced feeding activity in nearly all species (Scrimshaw 1991). Decreased food intake may be caused even by mild immune challenges, such as those associated with a simple vaccination (Gandra and Scrimshaw 1961). For example, female Eurasian blue tits *Cyanistes caeruleus* decreased feeding rates after vaccination with diphtheria (Råberg et al. 2000).

Adult birds can also respond to physiological stress by increasing foraging effort (Barbosa and Moreno 2004; Coon et al. 2011). However, increased feeding rates do not necessarily represent an increase in effective food provisioning by parents because the delivered food may be qualitatively poorer (Wright et al. 1998; Sanz et al. 2000). Immunised adults may feed their offspring also less selectively, as in the case of clipping feathers in adult great tits *Parus major* (both sexes), which initiated searching for food in the surrounding of their nest and provisioning food of poor quality to their offspring (Wegmann et al. 2015). Therefore, studies of food collected by the challenged parent birds should determine various food parameters, e.g. the number of food items and type and size of the food rather than merely the number of food items.

The amount of food may affect both cell-mediated immunity (measured by the wing web swelling after phytohaemagglutinin PHA injection) and humoral immunity (Bourgeon et al. 2006). In general, food-restricted birds have lower immunocompetence (Alonso-Alvarez and Tella 2001; Hangalapura et al. 2005; Brzęk and Konarzewski 2007). In the case of PHA-injected little ringed plovers *Charadrius dubius*, food-restricted birds had less wing web swellings in response to PHA than ad libitum fed birds (Gutiérrez et al. 2011). But in sand martins *Riparia riparia*, the PHA response was negatively correlated with the nestlings’ body mass increments when food was scarce and positively correlated when resources were plentiful (Brzęk and Konarzewski 2007). Therefore, I was interested in assessing also the diet of chicks in the context of resource allocation by parental birds during feeding.

The aim of this study was to examine whether food composition and immune condition of nestlings interact with the trade-off between parental reproductive effort and survival. In order to achieve this goal, female parents were injected with an antigen sheep red blood cells (SRBC). Females were chosen for the treatment because adult vertebrate males typically respond to immunological challenges less robustly than females (Nunn et al. 2009; Pap et al. 2010). I tested the following questions: (1) Does the parental reproductive effort (investment in offspring) decrease after immunisation with SRBC? (2) Does the parental investment in provisioning food to nestlings decrease in pairs, in which the female was experimentally immunised, as compared with control pairs? (3) Does diet composition provided to chicks change after maternal immunisation? (4) Do immunocompetence and body mass of chicks decrease after the challenge of the female’s immunity?

I predicted lower parental investment in terms of food provisioning rates to nestlings after immunisation of the female and lower body conditions of offspring, because females probably have to cope with the additional costs of the immune response, which may affect their parental investment. Consequences of the immune challenge on chick feeding were tested by assessing the volumes of food items and by identifying the different types of food provided by the parents to their nestlings 6 days after the female had been immunised.

## Materials and methods

### Study area

Research was conducted during two seasons: 2011 in Niepołomice Forest and 2012 in Krzyszkowice Forest (both near Kraków, Southern Poland), in populations of great tit *P. major* provided with nest boxes. In both locations, the distance between nest boxes was approximately 40–50 m; they were mounted about 2 m above the ground. The nest box area in Niepołomice Forest existed for 25 years and was based on 250 nest boxes. They were installed only in one isolated, most Northern part of the Niepołomice Forest called “Grobelczyk”, with a total area of 238 ha. The area including only trees with nest boxes (and 25-m buffers from each of them) was about 65 ha. The most common bird species occupying nest boxes was the great tit (about 70 nests per year). In 2011, breeding density of all birds breeding in the nest boxes (great tit, Eurasian blue tit, collared flycatcher *Ficedula albicollis*, European robin *Erithacus rubecula*) was 15.8 pairs per 10 ha. In Krzyszkowice Forest, 170 nest boxes were new and mounted before the breeding season in 2012, at the end of February. The whole forest area was 54.17 ha, including forest edges (bushes around woodland) and forest paths. About 45 ha contained rows of nest boxes with 25-m buffers around each of them. Breeding density of birds occupying nest boxes (great tits and Eurasian blue tits) was 18.1 pairs per 10 ha. In Krzyszkowice Forest, 45 broods of great tits were found. Both areas were deciduous forests dominated by oak-hornbeam, including oaks *Quercus robur* and *Q. petraea*, small-leaved lime *Tilia cordata*, hornbeam *Carpinus betulus* and European beech *Fagus sylvatica*. Niepołomice Forest was older woodland, with monumental trees. Krzyszkowice Forest was a younger “heterogeneous” complex, with more silver birch *Betula pendula* and Scots pine *Pinus sylvestris*.

### Field protocol

**Fig. 1:**
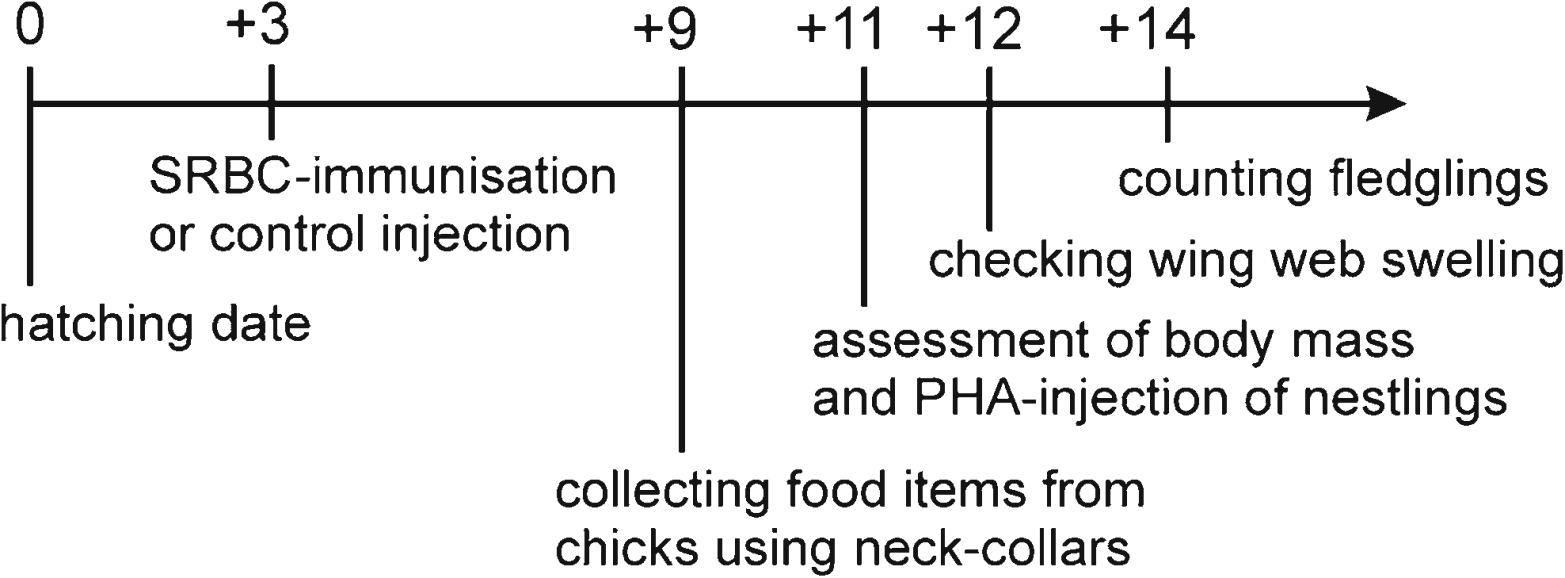
Timeline of experiment in both experimental (SRBC) and control (PBS) nests. *Horizontal line* is the time-axis; ticks are given for each day of the experiment, while labels (= treatments) are below the appropriate ticks

This study was conducted only on the first broods of great tits. Hatching date was the individual starting date (day 0 in Fig. 1) for procedures at the nests. Three days after hatching (+3 in Fig. 1), adults were captured using mist nets set up close to their nest boxes. In year 2011, adults were caught and recaptured in 19 nests, while in 2012 the experiment was performed at 24 nests. The antigen SRBC was used to stimulate a humoral immune response (Deerenberg et al. 1997; Cichoń et al. 2001; Hawley et al. 2005). In 21 nests (9 in 2011 and 12 in 2012), adult females were injected with 0.1 ml of sheep red blood cells (SRBC) as antigen in a 2% suspension with phosphate-buffered saline (PBS) following the protocols of Deerenberg et al. (1997) and Ros et al. (1997). In another 22 nests (10 in 2011 and 12 in 2012), control females were injected with plain PBS. All females were treated intraperitoneally with the antigen suspension (experimental group) or PBS (control group). During field work, the treatment (experimental/control) was assigned randomly to each female after both adult birds had been captured. The allocation of nests to the experimental and control groups was balanced between older and younger forest patches and across hatching dates to avoid effects due to the disparity of environmental factors between experimental groups.

Nine days after hatching (+9 in Fig. 1), when the maximum immune response to SRBC was expected (Snoeijs et al. 2007), food items were collected from nests. Soft collars made of velcros (Velcro, textile standard, black) were fastened around necks of nestlings for a constant 2-h period of time. The rings prevented the chicks from swallowing the food provided by their parents during 2 h. This allowed the experimenter to collect all caterpillars and other invertebrates (mainly spiders) from the bills of young birds using tweezers. The diet samples were stored in vials with 60% alcohol for later identification. The caterpillars found in the diet of great tit nestlings were butterfly larvae from three groups: noctuids Noctuidae, geometrids Geometridae and tortricids Tortricidae. In total, 270 food items were identified from *n* = 19 nests in the year 2011 and 306 food items from *n* = 21 nests were collected during the second season of a research (2012). The body shape of lepidopteran larvae resembles a cylinder. Similarly, the opisthosoma of spiders found in the nestling diet also had elongated cylindric shape. The lengths and widths of invertebrate food items were noted to calculate the volume (cm^3^) of each food item, which were then summarised per nest and used in analyses as “volume/size”. The volume of each food item was calculated using the formula for the volume of a simple circular cylinder (*V = π r* ^2^ *h*), where the measured length was the cylinder height (*h*), half of width was the radius of the cylinder base (*r*) and the value 3.14 was used for *π*. It was assumed that any minor bumps in the body fall under the limits of measurement error.

Two nests with SRBC females and one control nest from Krzyszkowice Forest (2012) were not included in the analyses of nestlings’ food composition, because the collars of at least one chick per nest had been removed (probably by adult birds). Neck collars are controversial from an ethical point of view. So far, there is no better and affordable method, which allows to collect invertebrates from food to identify quantitatively and qualitatively at the level of species for a constant unit of time. Faecal analysis is a much safer method, but it is unknown which period of time the collected food rested inside the digestive tract (Moreby and Stoate 2000), and stable isotope analysis is an expensive method (Pagani-Núñez et al. 2017). However, in this study all collared nestlings survived the treatment.

Eleven days after hatching (+11 in Fig. 1), the nestlings’ body mass was measured and they were treated with PHA for assessing their wing web swelling response. Chicks were weighted using an electronic scale MS 1000 (measurement accuracy *d* = 0.2 g). They were injected in the right wing web with 0.2 mg of phytohaemagglutinin (PHA, Sigma, L8754) diluted in 0.04 ml PBS (procedure according to Brinkhof et al. 1999; Cichoń et al. 2006). This substance is widely used as a non-pathogenic antigen, which provokes a T-cell-mediated immune reaction (Saino et al. 1997; Smiths et al. 1999; Salaberria et al. 2013). The PHA-induced immune reaction is a direct measure of the proliferative response of circulating T-lymphocytes combined with cytokines and inflammatory cells, which involves both the innate and adaptive components of immunity (Vinkler et al. 2010). In a wider viewpoint of immunology, the “immunocompetence” is the general capacity to respond to the antigen stimulation by an immune response (Cruse and Lewis 2009). From a practical point of view, immunocompetence is the effective immune response of the organism (Vinkler and Albrecht 2011). The presented test refers to the “wing web swelling” response in specific. To check whether the PHA antigen induced an immune response of chicks, the thickness of the treated nestling’s wing web was measured with a pressure-sensitive specimeter (Mitutoyo, 7313) around the area where the substance was injected. From each bird, these measurements were taken three times before the PHA injection (+11 in Fig. 1) and three times 24 h after the treatment (+12 in Fig. 1), when the maximum immune response was expected (Smiths et al. 1999; Salaberria et al. 2013). Then, the average of the three measures per bird was used in the analyses as a proxy of wing web swelling. For the purpose of PHA injection, I chose six nestlings per nest (two heaviest—in year 2011, mean ± sd was 15.11 ± 1.08 g, in 2012 16.91 ± 1.06; two medium-sized, in 2011 14.58 ± 0.89, in 2012 16.11 ± 0.93; and two lightest, in 2011 14.01 ± 0.92, in 2012 15.20 ± 1.18) to correct for the effect of body mass on the immune response. This selection was a compromise because I worked alone and there was no time and no need to treat all the chicks in a nest. However, the selective choice of chicks was probably a better measure of within-nest variation than an entirely random selection. The differences in chick sizes were very small (mean ± sd was approximately 1.76 g ± 0.78, based on data from 2 years), as the time window for hatching in Niepołomice and Krzyszkowice forests was narrow—it lasted 1–2 days, hence the asynchrony may be negligible. The final PHA experiment data set consisted of *n* = 19 nests from 2011 and *n* = 24 nests from 2012. Fourteen days after hatching (+14 in Fig. 1), the number of nestlings per nest box was reassessed to record fledgling success.

### Statistics

The impact of the experimental treatments and nesting parameters on the offspring wing web swelling and body mass was tested using four general linear mixed models in *JMP 8* (two GLMM models for each year of research). Distribution of each variable was checked also in *JMP* with Shapiro-Wilk test, which tests the null hypothesis that the sample comes from a normally distributed population. Distribution of wing web swelling of nestlings did not differ from normal distribution in year 2011 (*W* = 0.954; *P* = 0.212) and in 2012 (*W* = 0.989; *P* = 0.438), similarly as body mass was normally distributed both in 2011 (*W* = 0.986; *P* = 0.595) and in 2012 (*W* = 0.989; *P* = 0.334). GLMM models were based on data from 43 nests: 19 nests from 2011 (9 females were treated with SRBC) and 24 nests from 2012 (12 females were treated with SRBC). Data from individual nestlings were used as dependent variables, experimental treatment (SRBC or control) was a nominal factor, while nestling mass and number of nestlings were continuous explanatory variables. Nest identity (nest ID) was included as a random effect. Model parameters were estimated via restricted maximum likelihood (REML). Nestling body mass and numbers used in these models were measured on the day of offspring PHA immunisation prior to the injection.

Number of food items (caterpillars, spiders) and food volume were not normally distributed. According to the Shapiro-Wilk test, distribution of number of caterpillars from 2 years significantly differed from the normal distribution (*W* = 0.909; *P* < 0.0001), similarly as in the case of number of spiders (*W* = 0.909; *P* < 0.0001), number of all food items (*W* = 0.884; *P* = 0.007) and volume of food (*W* = 0.901; *P* = 0.019). Therefore, differences between food composition of nestlings in experimental (SRBC-immunisation) and control nests (PBS-injection) were compared with a non-parametric Mann-Whitney *U* test.

To test the influence of the number of caterpillars and spiders provided by adults on the wing web swelling and body mass of their nestlings, four generalised linear mixed models were performed in *JMP* for each experimental group (SRBC-experiment, PBS-control), separately. The dependent variables were nestling wing web swelling and individual body mass; nest ID was included as a random effect. Responses had a normal distribution of residuals; the chosen link function was identity, and the estimation method was REML. In each GLMM model, “year/forest” was treated as a nominal factor; nestling body mass and number of nestlings were continuous explanatory variables.

The fledgling success in nests of SRBC-treated mothers and nests of control mothers was compared with a *t* test for unpaired comparisons in *JMP* for each year of research, separately. Distribution of number of nestlings 14 days after hatching from 2 years did not differ from the normal distribution (*W* = 0.967; *P* = 0.234).

Results were considered significant with a probability of *P ≤* 0.050.

## Results

**Fig. 2:**
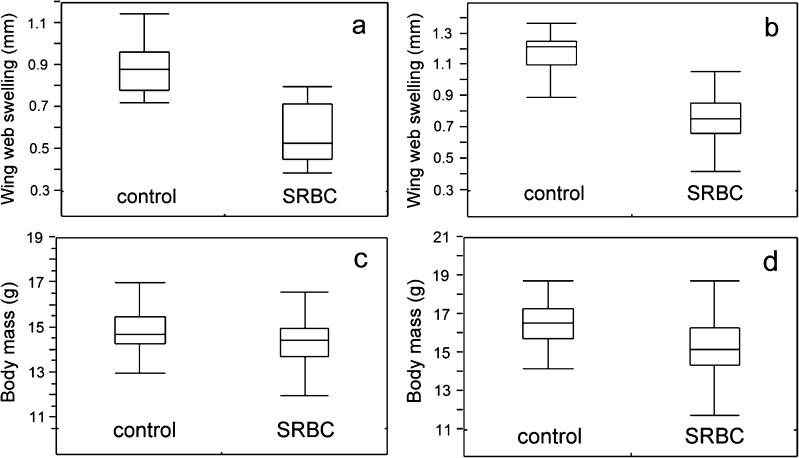
Nestling wing web swelling and body mass in the control (PBS) and experimental (SRBC) group, in 2011 (**a**, **c**) and 2012 (**b**, **d**). Each *box plot* shows the group 75th percentile, 25th percentile and median, while *whiskers* are maximum and minimum values

**Fig. 3:**
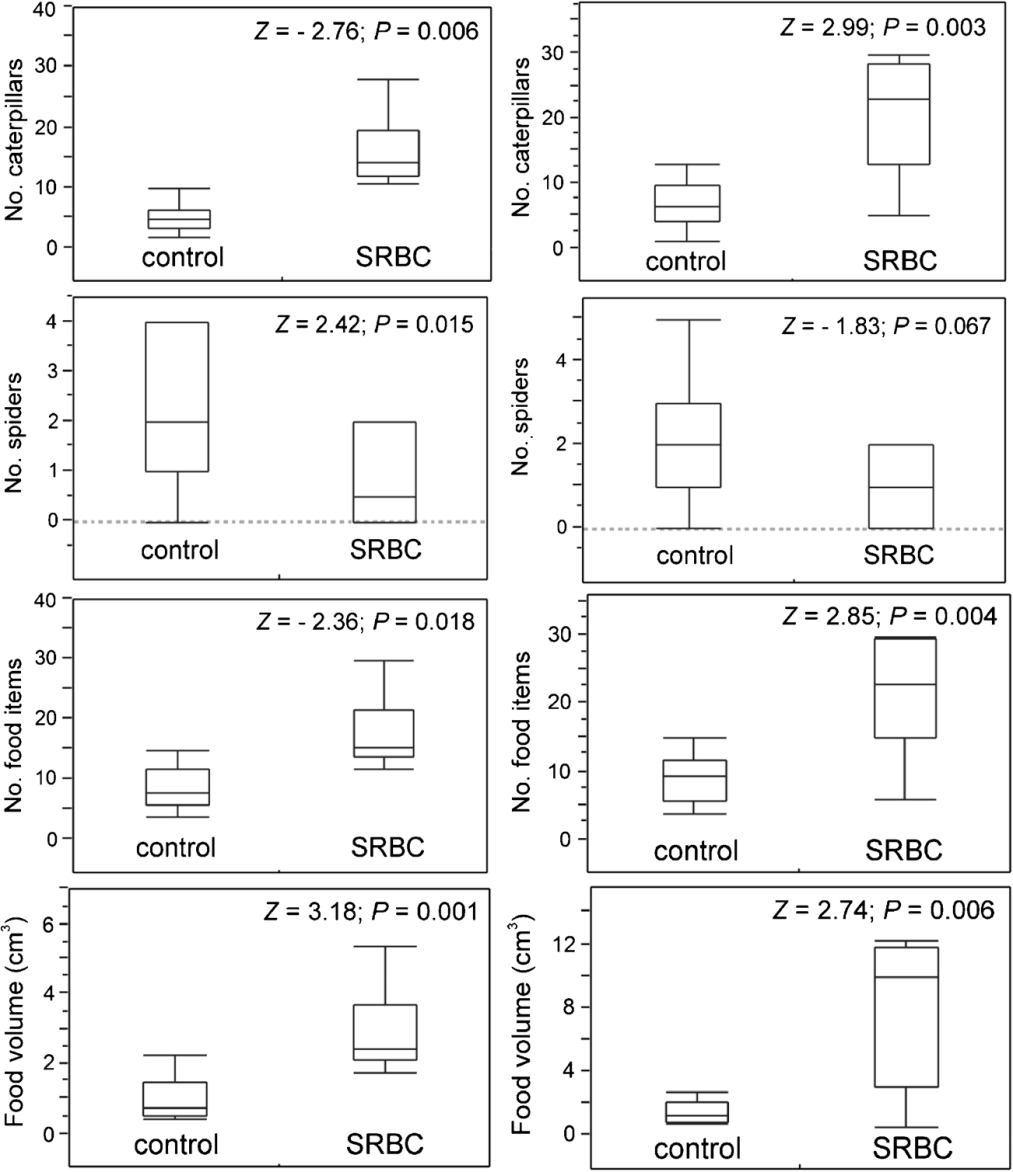
Number of caterpillars, spiders and all food items and volume of food provided by the parents to their offspring in the control (PBS) and the experimental (SRBC) groups. Above graphs are given results of Mann-Whitney *U* tests; in all cases *df* = 1. Each *box plot* shows the group 75th percentile, 25th percentile and median, while *whiskers* are maximum and minimum values

**Fig. 4:**
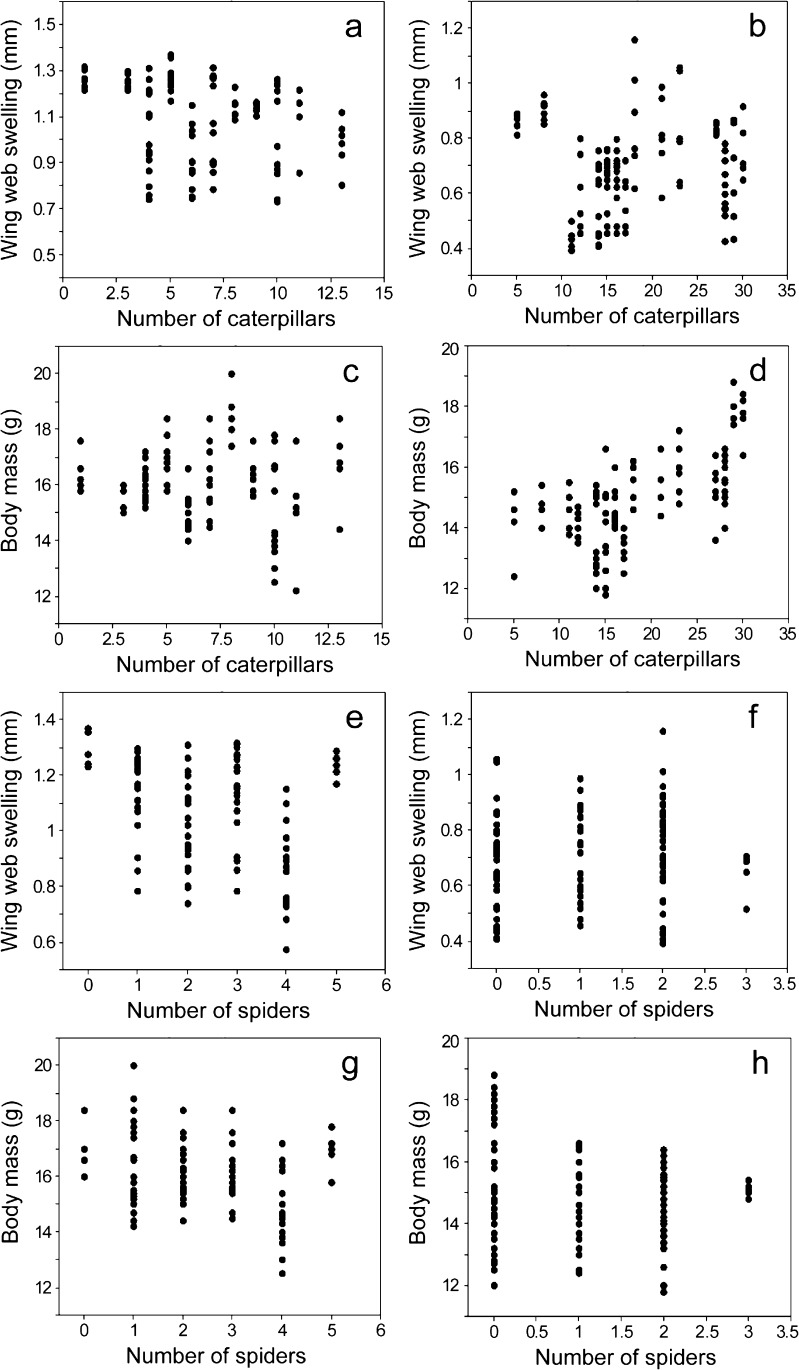
Relationships between chick diet composition in the case of rich food (caterpillars: **a**–**d**) and poor food (spiders: **e**–**h**) and their wing web swelling response to PHA injection and body mass in control (PBS) and experimental (SRBC) nests

The results of the GLMM analyses showed that nestling wing web swelling was lower in nests from SRBC-treated females than in those from control females both in 2011 and 2012, while body mass was lower in nests with the antigen immunisation than in control only in year 2012 (Tables 1 and 2, Fig. 2). SRBC-treated mothers provided more items, consisting of a higher proportion of caterpillars and fewer spiders with a higher total volume (Fig. 3) than females injected with PBS (control). Models designed for the control group showed that the number of caterpillars and spiders was negatively correlated with nestlings’ wing web swellings, while the number of spiders was also negatively associated with body mass of chicks (Table 3, Fig. 4). Models constructed for the experimental group (SRBC) showed no relationships between nestlings’ wing web swellings and diet, a positive correlation between offspring mass and number of caterpillars and a negative correlation between chick mass and number of spiders (Table 4, Fig. 4). Wing web swelling of nestlings was negatively correlated with number of nestlings both in nests from SRBC-treated females and in those of control females (Tables 3 and 4).

**Table 1:** Results of the GLMM models testing the influence of the experimental SRBC treatment of adult females on their nestlings’ wing web swelling and body mass (“nest ID” was treated as a random effect) in year 2011

Factors	*F*	*df*	*P*
Response: wing web swelling (*R* ^2^ = 0.676)			
Experiment	95.343	1	<0.0001
Body mass	1.909	1	0.179
Number of nestlings	0.063	1	0.807
Response: body mass (*R* ^2^ = 0.738)			
Experiment	1.036	1	0.331
Number of nestlings	0.612	1	0.451

**Table 2:** Results of the GLMM models testing the influence of the experimental SRBC treatment of adult females on their nestlings’ wing web swelling and body mass (“nest ID” was treated as a random effect) in year 2012

Factors	*F*	*df*	*P*
Response: wing web swelling (*R* ^2^ = 0.838)			
Experiment	64.878	1	<0.0001
Body mass	0.178	1	0.673
Number of nestlings	0.881	1	0.350
Response: body mass (*R* ^2^ = 0.706)			
Experiment	6.053	1	0.022
Number of nestlings	0.147	1	0.702

**Table 3:** Results of the models designed to test the effects of diet composition on the nestlings’ (*n* = 126) wing web swelling responses and body mass after PBS injection of the mothers (*n* = 21 treated adult females, control group)

Variables	*χ* ^2^	*df*	*P*
Response: wing web swelling			
Number of caterpillars	3.849	1	0.049
Number of spiders	4.685	1	0.030
Body mass	0.400	1	0.527
Number of nestlings	7.124	1	0.008
Year/forest	30.949	1	<0.0001
Response: body mass			
Number of caterpillars	1.513	1	0.219
Number of spiders	3.952	1	0.047
Number of nestlings	0.751	1	0.386
Year/forest	31.728	1	<0.0001

**Table 4:** Results of the models designed to test the effects of diet composition on the nestlings’ (*n* = 114 nestlings) wing web swelling response and body mass after SRBC injection of adult female (*n* = 19 treated adult females; experimental group)

Variables	*χ* ^2^	*df*	*P*
Response: wing web swelling			
Number of caterpillars	0.436	1	0.509
Number of spiders	0.558	1	0.455
Body mass	0.872	1	0.350
Number of nestlings	6.042	1	0.014
Year/forest	26.033	1	<0.0001
Response: body mass			
Number of caterpillars	32.502	1	<0.0001
Number of spiders	8.241	1	0.004
Number of nestlings	3.608	1	0.057
Year/forest	12.492	1	0.0004

In 2011, the number of fledged nestlings (+14 in Fig. 1) did not differ between nests from SRBC-treated mothers (mean ± se = 10.20 ± 0.39) and nests from control mothers (mean ± se = 9.67 ± 0.58; *t* = 0.77; *df* = 1; *P* = 0.456). Similarly, in 2012 the number of nestlings in experimental nests (mean ± se = 9.33 ± 0.50) was not significantly different from that in control nests (mean ± se = 10.17 ± 0.44; *t* = 1.25; *df* = 1; *P* = 0.228).

## Discussion

After the SRBC immune challenge of parental females, the nestlings’ wing web swellings (in response to PHA) were lower than in nestlings from the control group in both years of research. In 2012, also body mass of offspring was lower in experimental nests. The immune reaction of adult females probably caused physical discomfort; females decreased their investment and provided smaller amounts of food to their nestlings, which resulted in lower condition of chicks. Taking into account the fact that in great tits both sexes take care of the offspring, the changed quality and quantity of food items were probably related to both female and male behaviour; however, in this study focus was on the females and no data on the investment of male birds were collected. Certainly, the increased immune response of the female may have been important for the male’s immunity as it potentially affected males’ behaviour and investment in offspring. This study shows that immune responsiveness of parents is one of the key physiological traits negatively affecting their reproductive effort and survival. Organisms under nutrient limitation (such as the chicks in this study) have weaker immunity and become more susceptible to parasites and pathogen infections (Nelson and Demas 1996; Barbosa and Moreno 2004).

It is worth emphasising that the interpretation of the effects of any antigen injection may be complex, which should be taken into account also when interpreting the current results. For example, the production of antibodies may be energetically or nutritionally cheap, and producing high amounts of immunoglobulins might not constitute a major challenge for the organism. Also, the question remains to be answered whether a stronger antibody response reflects the better body condition of an animal, or whether the individual in better condition simply did not respond to the stimulation. Therefore, it may not be clear whether higher wing web swelling after PHA injection or stronger effort of adults after SRBC immunisation was a sign of a strong or conversely already challenged immune system. More resources provided per single chick could have affected their condition positively, including immunocompetence. In this work, chicks fed with more food after the experimental treatment had lower responses to PHA, which suggests their better condition. On the other hand, the offspring’s web swelling was negatively correlated with the number of chicks per nest, indicating that greater parental effort needed to take care of more chicks lowered their condition, suggesting a lower immune response of the weaker individuals. Therefore, a stronger response to PHA is regarded as characteristic for stronger birds rather than for those with weaker conditions.

According to the study of Krams et al. (2012), most common antigens that disable the quantification of the magnitude of immune response involve erythrocytes (e.g. sheep red blood cells—SRBC), toxic compounds of bacteria (e.g. lipopolysaccharide; tetanus and diphtheria toxins), plants (e.g. phytohaemagglutinin—PHA) and even animals (keyhole limpet haemocyanin). The diversity of antigens used in ecological research adds another problem to compare and interpret results (Norris and Evans 2000). It is also difficult to decide whether the results are caused by antigen properties or other factors, e.g. environmental conditions (Ots et al. 2001; Krams et al. 2012). The weaker response to PHA in the chicks after SRBC injection could have been due to their weakened condition, but later these chicks could have been also in better condition as a result of increased parental investment.

In pairs with experimentally immunised females, the parents (probably both) increased provisioning by increasing total food volume, number of food items provided to the nestlings and food quality—i.e. providing more caterpillars and fewer spiders. This change in quantity and quality of provided food might indicate the recovery of the parent and/or a response (of either mother or both parents) to the low nutritional status of their chicks. Higher amounts of caterpillars in the nestlings’ diet may be regarded as an increase in high-quality food. Caterpillars are relatively large food items, are rich in nutrients and had significant effects on chick body mass (Fig. 4d). One possible explanation is that studied birds increased feeding to cover the extraphysiological costs of immune defence. Under ideal circumstances, animals are fed and grown at an optimal rate, if there is no limit by a lack of resources (Metcalfe and Monaghan 2003). However, increased food intake allows to demonstrate an improved food conversion efficiency when submitted to an early-age food restriction, which was observed, for example, in broiler chickens (Jahanpour et al. 2013). “Compensatory growth” and “catch-up growth”—which are linked to compensatory feeding—are phenomena describing a faster than optimal growth occurring after a period of dietary restriction in many animals (Mangel and Munch 2005; Hector and Nakagawa 2012; Jahanpour et al. 2013). Compensatory growth refers to a faster than usual growth rate, while catch-up growth refers to delayed attainment of usual size (Hector and Nakagawa 2012).

It seems very likely that in the present study the increased intensity of feeding could be a phenomenon of compensatory feeding after a period of food restriction, which probably occurred after the immunisation of a female. There was a positive relationship between the number of caterpillars and nestling body mass in nests with SRBC injection, which may be explained by compensation feeding, although no relationship was observed between the number of provided caterpillars and wing web swelling response. This is not surprising, as also other authors found that higher food intake compensated for growth costs, but failed to compensate for the immunological cost, measured as T-cell-mediated immune response against an innocuous mitogen (Moreno-Rueda and Redondo 2012). It is possible that in the year 2011 a change in parental feeding was sufficient to compensate the mass of chicks in the experimental nests (with SRBC), and thus, no difference to the mass of young birds in the control nests was observed. Nevertheless, in the year 2012 lower body mass and immunocompetence of offspring in experimental than in control nests suggest that compensatory growth may require more than 8–9 days, which was the period between the experimental treatment of the female and PHA injection of chicks. It seems that the time to achieve the effect of compensatory feeding depends on external factors, such as environmental resources. Differences between forests in chick size and the amount of food provided by parents may explain significant effects of year/forest in the models calculated separately for each experimental group (control, SRBC).

The higher numbers of caterpillars in the chick diet at nests from immunised mothers (as compared with the control group) were accompanied by a lower proportion of spiders. Spiders contain lower levels of chitin than other food items, which makes them easy to digest by young birds (Magrath et al. 2004). Moreover, spiders include more taurine than caterpillars (Ramsay and Houston 2003), which is a protein important for the development of chick for bile, feathers and nervous system (Ramsay and Houston 2003). Earlier studies of other authors showed that in many passerines the diet of younger nestlings contains more spiders than that of older birds (Krebs and Avery 1984; Radford 2008). This confirms the importance of feeding also spiders during the initial stages of the nestlings’ development. However, in the present study the number of spiders was negatively correlated with nestling body mass in both experimental and control groups and with the nestlings’ wing web swelling response to PHA in control nests (PBS). The high proportion of caterpillars and the low number of spiders in the chick diet from experimental nests supports the concept of catch-up growth. Providing more caterpillars at the expense of spiders could have contributed to affecting the immunological status of young birds. In addition, spiders may contain ingredients important for the development of bird immunity.

The parent-offspring interactions also might explain parts of the observed variation. At the nests of immunised females, both parents provided more food to their offspring than parents at control nests. This is unexpected when the body mass of chicks was lower after SRBC treatment. These results also may have been due to increased begging rates of the chicks from immunised mothers. Begging is a conspicuous signal that indicates the need of nestlings for food (Kölliker et al. 1998; Loiseau et al. 2008). In many bird species, parents ignore begging chicks and even neglect smaller chicks that beg more (Caro et al. 2016). However, in this study similar numbers of fledged chicks were observed in experimental and control nests (without mortality after SRBC injection) and the chicks’ begging rates were not assessed.

Some authors previously explored the possibility that begging affects the immune function of birds (e.g. Buchanan et al. 2007; Soler et al. 2014; Redondo et al. 2016). Moreno-Rueda and Redondo (2011, 2012) found that intense begging reduced the immune function of Southern grey shrike *Lanius meridionalis* offspring. Begging may incur costs by reducing the nestlings’ immunocompetence (Moreno-Rueda 2010). Furthermore, body mass gain can be affected by the energetic cost of begging (Rodríguez-Gironés et al. 2001). Begging behaviour of nestlings may co-vary with nutritional needs (Wright et al. 2010) and affect the allocation of parental feedings (Gottlander 1987). In the present study, increased begging of chicks after the immunisation of the female parent may explain the lower immunocompetence in both years, body mass in year 2012 and higher parental provisioning (as compared with control nests). This interpretation is yet speculative and requires to be tested specifically in the future.

In conclusion, the results of the presented study showed changes in the food composition of chicks, their body mass (2012) and immunity (both years) after the mother’s immune system had been challenged with a novel antigen. In nests with experimentally treated mothers, both parents provided a higher volume of food, more food items and more caterpillars to their nestlings, whereas the number of spiders was lower than in control broods. This might be explained by parental compensational feeding after recovery from the inflammation of a female. Thus, the study shows in great tits that the trade-off between parental effort and survival of parents is mediated by the costs incurred for their immunity and can be assessed via food and condition of offspring.
